# Montreal Cognitive Assessment Hearing Impairment (MoCA-H) in brazilian portuguese: performance analysis

**DOI:** 10.1590/2317-1782/e20250242en

**Published:** 2026-07-06

**Authors:** Larianny Rutzen Lazzari, Rochele Martins Machado, Karina Carlesso Pagliarin, Fernanda Soares Aurélio Patatt

**Affiliations:** 1 Departamento de Fonoaudiologia, Universidade Federal de Santa Maria – UFSM - Santa Maria (RS), Brasil.

**Keywords:** Cognition, Hearing Loss, Psychometrics, Mental Status and Dementia Tests, Aged

## Abstract

**Purpose:**

To analyze the performance of the Montreal Cognitive Assessment Hearing Impairment (MoCA-H) in Brazilian Portuguese in neurologically healthy older adults, comparing those with normal hearing and those with hearing loss who use hearing aids.

**Methods:**

Observational, cross-sectional, and quantitative study involving 20 neurologically healthy older adults (with no signs of cognitive decline), matched by age and education, divided into two groups: one with a quadritonal average within normal limits, and the other with bilateral moderate or greater hearing loss, all users of bilateral Hearing Aids (HA). Participants were assessed through anamnesis, pure-tone audiometry, speech audiometry, tympanometry, Mini-Mental State Examination (MMSE), and MoCA-H. Comparisons between groups were conducted using Student’s t-test, with a significance level of 5%.

**Results:**

Similar performance was observed between groups in seven of the eight cognitive domains assessed by the MoCA-H. The only statistically significant difference was found in the visuo-spatial/executive domain, with lower performance in the group with hearing loss. No significant difference was found in the total MoCA-H score between the groups.

**Conclusion:**

Neurologically healthy older adults with and without treated hearing loss showed similar performance on the MoCA-H tasks. Only the visuospatial/executive task distinguished the groups evaluated. The MoCA-H proved to be an effective and sensitive tool in the cognitive assessment of older adults with moderate to severe hearing loss, at least in the sample studied.

## INTRODUCTION

The increase in life expectancy has contributed to the growth of the elderly population on a global scale, which brings new challenges to health systems, especially with regard to the preservation of cognitive and sensory functions^(1)^. Among the most common problems in this age group are hearing loss and cognitive decline, including dementia^(2)^. It is estimated that around 430 million people worldwide live with disabling hearing loss^(3)^, while approximately 55 million live with some type of dementia^(4)^.

Research indicates a relevant association between age-related hearing loss and an increased risk of cognitive decline, with hearing loss being a risk factor that contributes to the worsening of dementia cases^(5)^. Furthermore, the longer the period of hearing deprivation, the greater the increase in cognitive changes^(6,7)^, resulting in degradation of the neural system and reduction of its functions^(8)^.

Cognitive assessment, especially in older adults, is crucial for early identification of potential cognitive impairments and for preventing the worsening of conditions such as dementia^(9)^. To this end, various tests and screening tools have been developed and adapted to meet the specific characteristics of this population. In the case of individuals with hearing loss, limitations arise, since many conventional tests depend on verbal instructions, which can result in inaccurate diagnoses^(10-12)^.

Among the widely used screening tools is the Montreal Cognitive Assessment (MoCA)^(13)^, aimed at the early identification of mild cognitive impairment. However, its original version is not suitable for individuals with moderate or higher degrees of hearing loss, due to its dependence on auditory stimuli^(10)^.

Based on this limitation, an adapted version of the test was developed, the Montreal Cognitive Assessment Hearing Impairment (MoCA-H), which replaces verbal instructions with written visual stimuli, while maintaining the assessment of the same cognitive domains^(14)^. This adaptation aims to minimize the impact of hearing difficulties on understanding the instructions, although individual factors, such as the level of education, can still influence performance. The MoCA-H establishes a minimum criterion of four years of formal study for the participant to be able to answer it, and it is recommended to add one point to the final score for individuals with 12 years or less of schooling, according to the guidelines of the original instrument^(15)^.

Furthermore, regarding the reliability of the MoCA-H adaptation, the English version demonstrated a sensitivity of 92.8% and a specificity of 90.8%. Similar results were found in the German version, with a sensitivity of 97.5% and a specificity of 90%^(16)^, and in the Thai version, which obtained a sensitivity of 93.3% and a specificity of 80%^(17)^, reinforcing the applicability of the instrument in different cultural contexts.

More recently, the MoCA-H underwent a cross-cultural adaptation process for Brazilian Portuguese^(18)^ and the Brazilian version is now available on the official MoCA website. The linguistic and cultural adaptation of the MoCA-H for Brazilian Portuguese involved rigorous psychometric processes, including translation and back-translation of the protocol, analysis and selection of stimuli, semantic analysis of stimuli, analysis by expert and non-expert judges, as well as the pilot study, these steps being fundamental to ensure the validity and reliability of the tool in a new context^(18)^.

The use of these procedures follows international guidelines for adapting cognitive instruments, ensuring that the MoCA-H maintains its functional equivalence to the original version, without cultural or linguistic factors compromising its ability to adequately measure the proposed cognitive domains. However, more than just providing adapted versions, it is essential to understand the test's behavior in different user profiles. To this end, performance analysis by ability allows us to verify whether the test is behaving similarly between individuals with normal hearing and those with treated hearing loss, this investigation being essential to validate its effectiveness and clinical applicability.

Studies such as that of Völter et al.^(16)^, which investigated the German version of the MoCA-H, demonstrate that the instrument is suitable for people with hearing loss, showing high test-retest reliability and similar results between two groups, one with normal hearing or mild hearing loss, and the other with moderate to profound hearing loss, both groups without cognitive decline. These findings reinforce the importance of validating and applying the MoCA-H in different languages ​​and cultures, in order to ensure that the assessment takes into account cultural and linguistic impacts^(18)^ and is effectively focused on cognition, and not affected by sensory factors.

Given this, the present research is justified by the need to investigate whether the MoCA-H in Brazilian Portuguese can equivalently assess the cognitive performance of elderly individuals with and without treated hearing loss, contributing to the functional validation of the instrument and promoting greater accuracy in cognitive screenings in audiological clinical contexts. The objective of this study, therefore, was to analyze the performance of the MoCA-H in Brazilian Portuguese in two groups of neurologically healthy elderly individuals: one group with a quadritonal pure-tone average (4PTA) within normal limits and another with previously diagnosed hearing loss and hearing aid users. Based on this, it is expected that both groups will show similar performance in the skills assessed by the MoCA-H and in the instrument's total score, since they are neurologically healthy and do not present cognitive complaints.

## METHOD

This is an observational, cross-sectional, and quantitative study, with a sample selected by convenience, conducted in accordance with the guidelines and regulations for research involving human beings, as established in Resolution 466/12 of the National Health Council, and approved by the Research Ethics Committee (REC) of the institution where the study was developed, under number 5.162.650.

The current research was developed by the same group of researchers who carried out the cross-cultural adaptation of the Montreal Cognitive Assessment - Hearing Impairment (MoCA-H)^(18)^, as well as the criterion and construct validation stages of the instrument^(19)^. All individuals who consented to participate in the study signed the Informed Consent Form (ICF), which described the objective of the research, the procedures to which they would be subjected, as well as the risks and benefits involved.

Participants were recruited through the dissemination of the research on social networks, family and professional groups, as well as the participation of users already being monitored at the hearing aid clinic linked to a public university and at a private clinic in the interior of the state of Rio Grande do Sul (RS).

The eligibility criteria for the study were: age 60 years or older; absence of signs of cognitive decline, detected by applying the Mini-Mental State Examination - MMSE^(20)^; absence of self-reported previous neurological diseases; at least four years of formal education; preserved or corrected near visual acuity and, quadritonal pure-tone average (4PTA) (0.5kHz, 1kHz, 2kHz and 4kHz) within normality (<20dB) or moderate or higher degree of hearing loss, bilaterally^(3)^. For subjects with hearing loss, the following inclusion criteria were added: being fitted with hearing aids (HA) as indicated for each case, for at least three months and using the devices for at least six hours a day (verified by datalogging); and having open set speech comprehension, i.e., being able to recognize the word exclusively through hearing. Subjects who did not complete all the steps recommended by the study or who made it impossible to match the groups, considering the variables of age and education level, were excluded.

Based on the eligibility criteria, 30 participants were selected: 15 with a 4PTA within normal ranges and 15 with moderate or higher bilateral hearing loss (four-tone average equal to or greater than 35 dB, according to WHO, 2020). Of these, ten were excluded due to discrepancies in the data regarding the variables defined for matching.

The sample was initially divided, according to previously defined methodological criteria, into two distinct groups: Participants With No Hearing Loss (PNHL) and Participants With Hearing Loss (PWHL), the first consisting of elderly people with a 4PTA within the normal range (<20dB) and the second consisting of people who presented moderate or higher degree of hearing loss^(3)^.

After this division, the groups were matched by age and education level in order to ensure comparability between them and minimize possible biases related to these variables. Thus, the final sample consisted of 20 elderly individuals, equally distributed between the groups (ten in each) and between the sexes (ten male and ten female), with ages ranging from 61 to 74 years (A = 67.5; SD = 3.6) and formal schooling time between four and 25 years (A = 12.4; SD = 5.9) (Chart 1).

**Chart 1:** Characterization of the sample according to the variables age and education level

**Group**	**Subject**	**Age**	**Education (Years)**
**PNHL**	S1	66	15
	S2	64	13.5
	S3	68	17.5
	S4	64	17
	S5	64	17
	S6	65	16
	S7	65	5
	S8	70	21
	S9	70	11
	S10	66	11
**PWHL**	S11	68	13
	S12	67	5
	S13	73	17
	S14	70	8
	S15	73	11
	S16	68	5
	S17	70	8
	S18	61	7
	S19	63	4
	S20	74	25

**Caption:** PNHL – participants with no hearing loss; PWHL – participants with hearing loss

The characterization of the PWHL, regarding the type and degree of hearing loss, time of adaptation to hearing aids, and average daily use of the devices, is presented in Chart 2.

**Chart 2:** Characterization of PWHL regarding the type and degree of hearing loss, hearing aid experience and average daily hearing aid use

**Subject**	**Type of HL RE**	**Degree of HL RE**	**Type of HL LE**	**Degree of HL LE**	**Hearing aid experience**	**Datalogging h/day**
S11	SHL	Moderate	SHL	Moderately severe	9 months	14
S12	SHL	Moderately severe	SHL	Moderate	4 months	8
S13	SHL	Moderate	SHL	Moderate	7 months	11.8
S14	SHL	Severe	SHL	Moderate	3 months	13
S15	SHL	Moderately severe	SHL	Moderately severe	6 months	8
S16	SHL	Moderately severe	MHL	Moderately severe	3 months	10
S17	SHL	Severe	SHL	Moderately severe	11 months	7
S18	MHL	Severe	MHL	Severe	10 months	15.5
S19	SHL	Moderate	MHL	Moderately severe	15 months	6
S20	SHL	Complete	SHL	Moderate	6 months	6 in the best ear*

* Given that the right ear did not have amplifiable thresholds, the S20 prosthetic fitting was performed unilaterally.

**Caption:** PWHL – Participants with hearing loss; HL – hearing loss; SHL - Sensorineural hearing loss; MHL – Mixed hearing loss; RE - right ear; LE - left ear

For sample composition, the following procedures were performed: otoscopy, pure-tone audiometry, speech audiometry, immittance audiometry (tympanometry and acoustic reflex testing), application of the MMSE and case history. After this initial stage, participants were evaluated using the MoCA-H. The case history investigated the history and duration of hearing loss, presence of otitis media, habits such as headphone use, occupation and noise exposure, medical and family history (neurological or syndromic), medication use, possible visual impairments, and education level. Symptoms associated with COVID-19 infection were also investigated, including possible cognitive and/or memory complaints. However, in the absence of consistent cognitive complaints or a relevant family history, these reports were not further investigated, as there were no clinical indications to justify a more detailed inquiry.

Therefore, to assess cognitive functions and to define the study population, the MMSE was used. During the application of this instrument, all participants with hearing loss were using hearing aid and compensation strategies were employed (articulated speech, facing the subject, in a quiet and well-lit environment) whenever necessary, since its application is oral. The protocol has a total of 30 points, with normality standards based on schooling: 21 points - illiterate; 22 points - low schooling (one to five years); 23 points - medium schooling (six to 11 years); 24 points - high schooling (12 years or more)^(21)^.

Finally, the MoCA-H was administered by one of the two researchers who were properly trained to conduct and interpret the instrument, ensuring standardization and reliability in the procedures. The MoCA-H assesses cognitive functions through eight skills: visuospatial/executive skills, naming, memory, attention, language, abstract reasoning, delayed recall, and orientation.

The "visuospatial/executive" ability presents three tasks, totaling five points: one assesses cognitive flexibility and inhibition (executive functions) through an alternating trail test (score = 1); and two visuospatial tasks: copying a cube (score = 1) and drawing a clock (score = 3). "Naming" is assessed in the test based on the recall of three animal drawings, with each drawing corresponding to one point (score = 3). "Memory" is assessed using a list containing five words (face, velvet, roses, church, and blue), with instructions for the individual to repeat them in two attempts, even if successful on the first. At this point, no score is assigned. "Attention" is assessed through digit repetition in direct and reverse order (score = 2), mental calculation (score = 3), and vigilance (score = 1). The "language" skill is assessed through two tasks: verbal fluency, in which the participant must produce words beginning with the letter F in one minute (equal to or greater than 11 words, score = 1), and the rearrangement of words to construct sentences, with each correct sentence corresponding to one point (total score = 2). The "abstract reasoning" domain is assessed through the categorization of word pairs (score = 2). The "delayed recall" skill consists of remembering the list of words presented previously in the "memory" skill (face, velvet, roses, church, and blue), with each correctly recalled word corresponding to one point (total score = 5). Finally, the "orientation" domain is assessed by the participant's ability to state the date (score = 1), month (score = 1), year (score = 1), day of the week (score = 1), location (score = 1), and city (score = 1) where they are located, totaling 6 points.

For the application of this instrument, 77 cards (provided on an A4 sheet, in landscape orientation) containing the activities that make up the test were presented. The participant read each card aloud and had to follow the instructions on it. In the first three tasks of the test, the individual was asked, using the instructions on the cards, to manually solve some tasks; at this point, they were given the test sheet and a pen. The time used for the application of the protocol was 20 to 30 minutes, varying according to difficulties related to the subjects' schooling and reading and writing habits, which were not formally assessed, only self-reported by the participants. Even so, there was no assistance with reading from the researchers, since this intervention is not foreseen in the procedure. It is emphasized that, under no circumstances should one interfere in the application of the instrument. The assessments were carried out individually, in quiet rooms, in a single meeting of approximately two hours.

The assessment data were tabulated and statistical analyses were conducted using Jamovi software (version 2.6.45.0). The normality of the variables was verified using the Shapiro-Wilk test. Most variables showed a non-normal distribution (p < 0.05), except for the Total MoCA. Therefore, the non-parametric Mann-Whitney test was used for comparisons between groups. For the total MoCA variable, the parametric Student's t-test was used. The significance level adopted was 5%.

## RESULTS

Table 1 presents the average performance scores on the different tasks of the MoCA-H, comparing the results obtained by participants in the hearing loss group with those in the group without hearing loss.

**Table 1:** Comparison of skills assessed in the MoCA-H between groups with and without hearing loss

**Task/maximum score**	**Group**	**N**	**Average**	**Standard Deviation**	**p-value**
Visuospatial/executive/5	PWHL	10	2.7	0.8	0.012
	PNHL	10	3.9	1.1	
Naming/3	PWHL	10	2.6	0.5	0.681
	PNHL	10	2.7	0.5	
Memory	PWHL	10	7.4	1.3	0.331
	PNHL	10	8.1	1.2	
Attention/6	PWHL	10	3.8	1.4	0.436
	PNHL	10	4.3	1.5	
Language/3	PWHL	10	1.4	1.2	0.081
	PNHL	10	2.3	0.9	
Abstract reasoning/2	PWHL	10	0.8	0.9	0.468
	PNHL	10	1.1	0.9	
Delayed recall/5	PWHL	10	2.3	1.4	0.482
	PNHL	10	2.7	1.6	
Orientation/6	PWHL	10	5.8	0.4	0.185
	PNHL	10	5.5	0.5	
MoCA-H Total/30	PWHL	10	20.8	2.6	0.123
	PNHL	10	23.1	4.2	

Statistical test: Student's t-test and Mann-Whitney test

**Caption:** PWLH – Participants with hearing loss; PNHL – Participants with no hearing loss

From the results (Table 1), it was observed that, in general, both groups showed similar performance on the tasks that make up the MoCA-H, with no statistically significant differences in most of the skills assessed (naming, memory, attention, language, abstract reasoning, delayed recall, and orientation), including the total MoCA-H score. The only significant difference was identified in the visuospatial/executive domain (p = 0.012), in which the PWHL showed a lower average (A = 2.7; SD = 0.8) compared to the PNHL (A = 3.9; SD = 1.1).

## DISCUSSION

The performance analysis phase aimed to verify the functioning of the items of the instrument studied between the two groups, with the expectation that they would present similar results. Considering that, at this point in the research, the participants should not show signs of cognitive decline identified by the MMSE, the groups were organized in order to maintain equivalence in the variables of age and education, differing essentially in the presence or absence of hearing loss.

Furthermore, the effective use of individual hearing aids (HA) was established as eligibility criteria for the group with hearing loss. The use of HAs is recognized for promoting better sensory adaptation and integration of auditory information, reducing the cognitive load required for understanding linguistic stimuli^(22)^. These benefits occur, in part, because the amplification devices minimize the auditory effort required to decode speech, allowing cognitive resources to be directed more efficiently to processing and understanding the information received^(23,24)^.

Regular use of hearing aids has been associated with a lower rate of cognitive decline over time, especially when compared to individuals who do not treat hearing loss^(25)^. Furthermore, studies indicate that the use of these devices contributes to the maintenance of specific cognitive functions, such as working memory and attention, reinforcing their role in preserving cognition in older adults^(26)^.

In this context, the groups of elderly people with and without hearing loss showed similar performance in seven of the eight domains assessed by the MoCA-H. This finding was expected, since the instrument was specifically adapted for individuals with hearing impairment, eliminating the need for auditory comprehension during its application and thus reducing the impact of hearing loss on the assessment of cognitive functions^(13,14)^.

Similarly, a previous study that applied the MoCA-H to hearing aid users demonstrated that visual adaptation of the test reduces the influence of hearing loss on cognitive performance, enabling a more accurate identification of cognitive deficits^(17)^. This result can be explained by the fact that the use of the MoCA-H neutralizes one of the main biases in cognitive assessment: auditory perceptual overload and difficulty in understanding verbal commands presented orally^(10)^.

However, despite the similarity in overall performance between the groups, a significant difference was found in the visuospatial/executive task, requiring further investigation into the factors associated with this result. This finding corroborates the findings of the German adaptation of the instrument, which also identified significant differences in this ability when comparing two groups: one consisting of individuals with hearing thresholds within normal limits to mild hearing loss and another consisting of individuals with moderate to profound hearing loss^(16)^. These results suggest that hearing loss can negatively impact the visuospatial/executive domain.

Regarding the possible justifications for the observed difference, it is noteworthy that, in elderly individuals with hearing loss, there is evidence of structural and functional changes in the brain that can affect visuospatial and executive cognitive abilities. The study by Kirschen and Leaver^(27)^ used neuroimaging to show that higher hearing thresholds are associated with a reduction in white matter volume in temporal and parahippocampal regions, changes in cortical thickness, and ventricular expansion, suggesting brain reorganization or degeneration related to hearing loss. In addition to volumetric changes, hearing loss can induce neuronal deafferentation and functional reorganization of the auditory cortex. Dietrich et al.^(28)^ demonstrated, using magnetoencephalography in adults with high-frequency hearing loss, that deafferentation resulting from cochlear damage promotes cortical reorganization in auditory areas, altering patterns of functional activation and recruitment of adjacent regions. In a more recent study, Tong et al.^(29)^ used advanced functional connectivity techniques to map the functional gradient of the cortical connectome in older adults with age-related hearing loss, showing redistribution of activity in higher sensory and cognitive networks, suggesting that cortical reorganization is not restricted to auditory areas, but involves integrated changes in multiple neural networks. These findings indicate that auditory deafferentation can impact the efficiency of brain networks associated with complex cognitive functions, such as visuospatial processing and the execution of higher-order cognitive tasks.

Furthermore, when comparing magnetic resonance imaging between individuals with hearing loss and individuals with normal hearing, Lin et al.^(30)^ identified a greater reduction in total brain volume and also a greater reduction in the volume of the right temporal lobe among individuals with hearing loss, reinforcing the association between hearing deficits and brain structural changes. Corroborating these findings, a prospective cohort study conducted by Armstrong et al.^(31)^ pointed to acquired hearing loss in adulthood as a risk factor for volumetric reduction of the temporal lobe at older ages. Considering that the temporal lobe is responsible for essential functions such as language processing, memory, auditory processing and visual skills, including recognition of visual patterns, faces and familiar objects, these structural changes may have significant implications for the individual's visuospatial and oculomotor skills^(32)^. Also, because of this, individuals without hearing loss tend to perform better in visuospatial skills and executive functions, possibly due to the integrity of the auditory system, the temporal lobe and neural pathways, since hearing plays a crucial role in the development and maintenance of cognitive functions, acting as a fundamental input pathway for environmental stimuli that promote neural plasticity and efficient cognitive functioning^(33)^.

Another possible hypothesis for the lower performance of participants with hearing loss in the visuospatial/executive task may be related to the order in which the activities were applied in the MoCA-H. Considering that the lower performance was observed only in the first task, it is possible that anxiety or initial excitement negatively influenced the results. In this context, Yerkes-Dodson's Law can be applied, since it describes how high levels of emotional arousal at the beginning of a task can impair performance, especially in activities that require greater cognitive control^(34)^.

According to the theory, the start of an assessment with more challenging tasks raises the individual's arousal levels to the point of exceeding the optimal performance point, resulting in below-expected performance. The anxiety generated by the need to adapt quickly to a more complex task consumes cognitive resources and generates a temporary cognitive overload, impairing the ability to concentrate and perform the task effectively. This leads to a decrease in performance, which can be overcome in subsequent tasks as arousal levels stabilize^(35)^, although this effect can be observed generally in different populations, not being exclusive to individuals with hearing loss.

Furthermore, the complexity of these tasks can also be an influential factor, since in parallel with the process of the emergence of age-related hearing changes, older adults often face additional difficulties in complex cognitive tasks, due to the increased cognitive load required to compensate for sensory loss^(7,36)^. The decline in cognitive functions can result in less availability of resources for performing activities that require planning, organization, comprehension, problem-solving and memory, functions typically associated with the prefrontal areas of the brain^(37)^.

A possible methodological bias to be considered relates to the planning of data collection, since the entire battery of examinations and tests, including directed anamnesis, reading and signing of the Informed Consent Form, pure-tone audiometry, speech audiometry, immittance audiometry (tympanometry and acoustic reflex testing), MMSE and MoCA-H, was performed on the same day. The MMSE and MoCA-H cognitive protocols were always applied last, although, in some cases, there was variation in the order between the two instruments. This strategy may have led to cognitive fatigue in the participants, which, in turn, may have impacted performance on the tests, considering that recent studies indicate that prolonged cognitive tasks can induce mental fatigue, negatively affecting cognitive and physical performance in older adults^(38)^.

An additional limitation relates to the educational profile of the participants, whose average schooling was 12.4 years (SD = 5.9), higher than the national average for people aged 25 or over in 2024, according to IBGE data^(39)^. Although the national average has consistently increased, from 9.1 years in 2016 to 10.1 years in 2024, the schooling of the study sample still remains above this average, and it is important to note that the results found may not fully reflect the reality of older people with lower schooling or from regions with different educational profiles.

Furthermore, administering the tests at the end of an extensive battery can generate impatience or haste to complete the procedure, compromising participant engagement and concentration^(40)^. The possible anxiety generated by the duration of the assessment or the complexity of the final tasks may also have influenced performance, especially in the visuospatial/executive task of the MoCA-H, which was the first presented in the protocol, as discussed based on Yerkes-Dodson's Law^(35)^. In short, the significant difference observed in the visuospatial/executive task between elderly individuals with and without hearing loss can be explained by several factors, such as neuronal deafferentation, the order of activities in the test, and the complexity of the task.

Considering the overall results, the absence of significant differences in seven of the eight assessed abilities, as well as in the total MoCA-H score, suggests that this instrument is effective in evaluating cognitive functions in older adults with hearing loss, minimizing the impact of the sensory impairment on the results. This finding reinforces the importance of using adapted instruments, such as the MoCA-H, which ensure a more accurate and equitable assessment of cognitive performance in clinical and research contexts. By eliminating barriers related to auditory comprehension, the MoCA-H allows the cognitive abilities of older adults to be assessed more reliably, offering a valuable tool for the early detection of cognitive impairments, without the bias of hearing loss.

## CONCLUSION

It was found that neurologically healthy elderly individuals, both with and without treated hearing loss, showed similar performance in most of the MoCA-H tasks. Only the visuospatial/executive domain significantly differentiated the groups evaluated. Overall, the MoCA-H proved to be an effective and sensitive tool for the cognitive assessment of elderly individuals with moderate to severe hearing loss, at least in the sample analyzed.

## References

[1] de Azeredo Passos VM, Champs APS, Teixeira R, Lima-Costa MFF, Kirkwood R, Veras R (2020). The burden of disease among Brazilian older adults and the challenge for health policies: results of the Global Burden of Disease Study 2017. Popul Health Metr.

[2] Pereira WAB, de Oliveira IR, Werner F, dos Santos LT, Furtado PS, Brigagão RV (2023). Aumento da expectativa de vida e crescimento populacional no Brasil e os impactos no número de pessoas vivendo com doenças crônico-degenerativas: desafios para o manejo da Doença de Alzheimer. Res Soc Dev.

[3] WHO: World Health Organization (2021). World report on hearing.

[4] WHO: World Health Organization (2021). Dementia.

[5] Loughrey DG, Kelly ME, Kelley GA, Brennan S, Lawlor BA (2018). Association of age-related hearing loss with cognitive function, cognitive impairment, and dementia: a systematic review and meta-analysis. JAMA Otolaryngol Head Neck Surg.

[6] Lin FR, Metter EJ, O’Brien RJ, Resnick SM, Zonderman AB, Ferrucci L (2011). Hearing loss and incident dementia. Arch Neurol.

[7] Lin FR, Yaffe K, Xia J, Xue QL, Harris TB, Purchase-Helzner E (2013). Hearing loss and cognitive decline in older adults. JAMA Intern Med.

[8] Sharma A, Glick H (2016). Cross-modal re-organization in clinical populations with hearing loss. Brain Sci.

[9] Tsoi KKF, Chan JYC, Hirai HW, Wong SYS, Kwok TCY (2015). Cognitive Tests to Detect Dementia: A Systematic Review and Meta-analysis. JAMA Intern Med.

[10] Dupuis K, Pichora-Fuller MK, Chasteen AL, Marchuk V, Singh G, Smith SL (2015). Effects of hearing and vision impairments on the Montreal Cognitive Assessment. Neuropsychol Dev Cogn B Aging Neuropsychol Cogn.

[11] Jorgensen LE, Palmer CV, Pratt S, Erickson KI, Moncrieff D (2016). The Effect of Decreased Audibility on MMSE Performance: A Measure Commonly Used for Diagnosing Dementia. J Am Acad Audiol.

[12] Völter C, Götze L, Dazert S, Wirth R, Thomas JP (2020). Impact of Hearing Loss on Geriatric Assessment. Clin Interv Aging.

[13] Nasreddine ZS, Phillips NA, Bédirian V, Charbonneau S, Whitehead V, Collin I (2005). The Montreal Cognitive Assessment, MoCA: a brief screening tool for mild cognitive impairment. J Am Geriatr Soc.

[14] Dawes P, Reeves D, Yeung WK, Holland F, Charalambous AP, Côté M (2023). Development and validation of the Montreal cognitive assessment for people with hearing impairment (MoCA-H). J Am Geriatr Soc.

[15] Nasreddine Z (2017). Montreal Cognitive Assessment (MoCA) Administration & Scoring Instructions (Version 8.1): Montreal Cognitive Assessment.

[16] Völter C, Fricke H, Götze L, Labrenz F, Tokic M, Wirth R (2022). Evaluation of the non-auditory neurocognitive test MoCA-HI for hearing-impaired. Front Neurol.

[17] Utoomprurkporn N, Stott J, Costafreda SG, North C, Heatley M, Bamiou DE (2021). The screening accuracy of a visually based Montreal cognitive assessment tool for older adult hearing aid users. Front Aging Neurosci.

[18] Machado RM, Pagliarin KC, Patatt FSA (2025). Montreal Cognitive Assessment Hearing Impairment (MoCA-H): cross-cultural adaptation to Brazilian Portuguese. CoDAS.

[19] Konrath G, Machado RM, Pagliarin KC, Patatt FSA (2025). Montreal Cognitive Assessment Hearing Impairment (MoCA-H) no Português Brasileiro: validade de critério e de construto. CoDAS.

[20] O’Driscoll C, Shaikh M (2017). Cross-Cultural Applicability of the Montreal Cognitive Assessment (MoCA): a systematic review. J Alzheimers Dis.

[21] Chaves MLF, Izquierdo I (1992). Differential diagnosis between dementia and depression: a study of efficiency increment. Acta Neurol Scand.

[22] Pichora-Fuller MK, Kramer SE, Eckert MA, Edwards B, Hornsby BW, Humes LE (2016). Hearing Impairment and Cognitive Energy: the Framework for Understanding Effortful Listening (FUEL). Ear Hear.

[23] Lunner T, Rudner M (2013). Cognitive spare capacity as a window on hearing aid benefit. Semin Hear.

[24] Peelle JE (2018). Listening effort: how the cognitive consequences of acoustic challenge are reflected in brain and behavior. Ear Hear.

[25] Maharani A, Dawes P, Nazroo J, Tampubolon G, Pendleton N, SENSE-Cog WP1 group (2018). Longitudinal relationship between hearing aid use and cognitive function in older americans. J Am Geriatr Soc.

[26] Sarant J, Harris D, Busby P, Maruff P, Schembri A, Lemke U (2020). The effect of hearing aid use on cognition in older adults: can we delay decline or even improve cognitive function?. J Clin Med.

[27] Kirschen RM, Leaver AM (2024). Hearing function moderates age-related differences in brain morphometry in the HCP aging Cohort. Hum Brain Mapp.

[28] Dietrich V, Nieschalk M, Stoll W, Rajan R, Pantev C (2001). Cortical reorganization in patients with high frequency cochlear hearing loss. Hear Res.

[29] Tong Z, Zhang J, Xing C, Xu X, Wu Y, Salvi R (2023). Reorganization of the cortical connectome functional gradient in age-related hearing loss. Neuroimage.

[30] Lin FR, Ferrucci L, An Y, Goh JO, Doshi J, Metter EJ (2014). Association of hearing impairment with brain volume changes in older adults. Neuroimage.

[31] Armstrong NM, An Y, Doshi J, Erus G, Ferrucci L, Davatzikos C (2019). Association of midlife hearing impairment with late-life temporal lobe volume loss. JAMA Otolaryngol Head Neck Surg.

[32] Clarke A, Federmeier KD, Beck DM (2019). Psychology of learning and motivation.

[33] Samelli AG, Gonçalves NG, Padilha FYOMM, Guesser VM, Matas CG, Rabelo CM (2025). Hearing loss and cognitive decline in the Brazilian Longitudinal Study of Adult Health (ELSA-Brasil) during eight years of follow-up. J Alzheimers Dis.

[34] Mair RG, Onos KD, Hembrook JR (2011). Cognitive activation by central thalamic stimulation: the yerkes-dodson law revisited. Dose Response.

[35] Yerkes RM, Dodson JD (1908). The relation of strength of stimulus to rapidity of habit formation. J Comp Neurol Psychol.

[36] Cardoso MJF, Alvarenga KF, Tabaquim MLM, Lopes TA, Costa AO, Jacob LCB (2024). Idosos com perda auditiva e declínio cognitivo: desempenho da percepção de fala no ruído. CoDAS.

[37] Peelle JE, Wingfield A (2016). The neural consequences of age-related hearing loss. Trends Neurosci.

[38] Martin K, Meeusen R, Thompson KG, Keegan R, Rattray B (2018). Mental fatigue impairs endurance performance: a physiological explanation. Sports Med.

[39] IBGE: Instituto Brasileiro de Geografia e Estatística (2025). Indicadores educacionais avançam em 2024, mas atraso escolar aumenta.

[40] Ackerman PL, Kanfer R (2009). Test length and cognitive fatigue: an empirical examination of effects on performance and test-taker reactions. J Exp Psychol Appl.

