# Supplementary material for: Montreal Cognitive Assessment Hearing Impairment (MoCA-H) in brazilian portuguese: performance analysis
Source: Codas. 2026 Jul 6;38(4):e20250242. doi: 10.1590/2317-1782/e20250242en (PMC13343790; doi:10.1590/2317-1782/e20250242en)
Supplement: Translation

# Translation: Montreal Cognitive Assessment Hearing Impairment (MoCA-H) no português brasileiro: análise de desempenho

**DOI:** 10.1590/2317-1782/e20250242pt

**Authors:** Larianny Rutzen Lazzari, Rochele Martins Machado, Karina Carlesso Pagliarin, Fernanda Soares Aurélio Patatt

**Affiliations:** 1 Departamento de Fonoaudiologia, Universidade Federal de Santa Maria – UFSM - Santa Maria (RS), Brasil.

**Keywords:** Cognição, Perda Auditiva, Psicometria, Testes de Estado Mental e Demência

## Abstract

**Objetivo:**

Analisar o desempenho do Montreal Cognitive Assessment Hearing Impairment (MoCA-H) em português brasileiro em idosos neurologicamente saudáveis, comparando aqueles com audição normal e com perda auditiva usuários de próteses auditivas.

**Método:**

Estudo observacional, transversal e quantitativo, com 20 idosos neurologicamente saudáveis (sem sinais de declínio cognitivo), pareados por idade e escolaridade, divididos em dois grupos: um com média quadritonal dentro da normalidade e outro com perda auditiva bilateral de grau moderado ou superior, usuários de Aparelhos de Amplificação Sonora Individual (AASI). Os participantes foram avaliados por meio de anamnese, audiometria tonal liminar, logoaudiometria, imitanciometria, Mini Exame do Estado Mental (MEEM) e MoCA-H. As comparações entre os grupos foram realizadas com o teste t de Student e Mann Whitney com nível de significância de 5%.

**Resultados:**

Observou-se desempenho semelhante entre os grupos em sete das oito habilidades avaliadas pelo MoCA-H. A única diferença estatisticamente significativa foi identificada no domínio visuo-espacial/executivo, com desempenho inferior no grupo com perda auditiva. A pontuação total do MoCA-H não apresentou diferença significativa entre os grupos.

**Conclusão:**

Os idosos neurologicamente saudáveis sem e com perda auditiva tratada apresentaram desempenho semelhante nas tarefas do MoCA-H. Apenas a tarefa visuo-espacial/executivo distinguiu os grupos avaliados. O MoCA-H mostrou-se uma ferramenta eficaz e sensível na avaliação cognitiva de idosos com perda auditiva moderada à severa, ao menos na amostra estudada.

## INTRODUÇÃO

O aumento da expectativa de vida tem contribuído para o crescimento da população idosa em escala global, o que traz novos desafios para os sistemas de saúde, especialmente no que diz respeito à preservação das funções cognitivas e sensoriais^(1)^. Entre os agravos mais comuns nessa faixa etária, destacam-se a perda auditiva e o declínio cognitivo, incluindo os quadros demenciais^(2)^. Estima-se que cerca de 430 milhões de pessoas no mundo vivam com perda auditiva incapacitante^(3)^, enquanto aproximadamente 55 milhões convivem com algum tipo de demência^(4)^.

Pesquisas indicam uma associação relevante entre a perda auditiva relacionada ao envelhecimento e o risco elevado de declínio cognitivo, sendo a perda auditiva um fator de risco que contribui para o agravamento dos casos demenciais^(5)^. Além disso, quanto maior o tempo de privação auditiva, maior o aumento das alterações cognitivas^(6,7)^, resultando em degradação do sistema neural e redução das suas funções^(8)^.

A avaliação da cognição, especialmente em idosos, é crucial para identificar precocemente possíveis comprometimentos cognitivos e prevenir o agravamento de condições como a demência^(9)^. Para isso, diversos testes e ferramentas de triagem têm sido desenvolvidos e adaptados, com o intuito de atender às características específicas dessa população. No caso de indivíduos com perda auditiva, surgem limitações, uma vez que muitos testes convencionais dependem de instruções verbais, o que pode resultar em diagnósticos imprecisos^(10-12)^.

Entre as ferramentas de triagem amplamente utilizadas está o Montreal Cognitive Assessment (MoCA)^(13)^, voltado à identificação precoce de comprometimento cognitivo leve. Contudo, sua versão original não é adequada para indivíduos com deficiência auditiva de grau moderado ou superior, devido à sua dependência de estímulos auditivos^(10)^.

A partir dessa limitação, foi desenvolvida uma versão adaptada do teste, o Montreal Cognitive Assessment Hearing Impairment (MoCA-H), que substitui instruções verbais por estímulos visuais escritos, mantendo a avaliação dos mesmos domínios cognitivos^(14)^. Essa adaptação visa minimizar o impacto de dificuldades auditivas na compreensão das instruções, embora fatores individuais, como o nível de escolaridade, ainda possam influenciar no desempenho. O MoCA-H estabelece um critério mínimo de quatro anos de estudo formal para que o participante consiga respondê-lo, sendo recomendada a adição de um ponto à pontuação final em indivíduos com 12 anos ou menos de escolaridade, conforme a orientação do instrumento original^(15)^.

Ainda, sobre a fidedignidade da adaptação do MoCA-H, a versão em inglês demonstrou sensibilidade de 92,8% e especificidade de 90,8%. Resultados semelhantes foram encontrados na versão alemã, com sensibilidade de 97,5% e especificidade de 90%^(16)^, e na versão tailandesa, que obteve sensibilidade de 93,3% e especificidade de 80%^(17)^, reforçando a aplicabilidade do instrumento em diferentes contextos culturais.

Mais recentemente, o MoCA-H passou por um processo de adaptação transcultural para o português brasileiro^(18)^ e a versão brasileira já se encontra disponível no site oficial do MoCA. A adaptação linguística e cultural do MoCA-H para o português brasileiro envolveu rigorosos processos psicométricos, incluindo tradução e retrotradução do protocolo, análise e seleção dos estímulos, análise semântica dos estímulos, análise de juízes especialistas e não especialistas, além do estudo piloto, sendo estas etapas fundamentais para assegurar a validade e a confiabilidade da ferramenta em um novo contexto^(18)^.

A utilização desses procedimentos segue as diretrizes internacionais para adaptação de instrumentos cognitivos, garantindo que o MoCA-H mantenha sua equivalência funcional em relação à versão original, sem que fatores culturais ou linguísticos comprometam sua capacidade de mensurar adequadamente os domínios cognitivos propostos. No entanto, mais do que disponibilizar versões adaptadas, é essencial compreender o comportamento do teste em diferentes perfis de usuários. Para isso, a análise do desempenho por habilidades permite verificar se o teste está se comportando de maneira semelhante entre indivíduos com audição normal e aqueles com perda auditiva tratada, sendo esta investigação essencial para validar sua eficácia e aplicabilidade clínica.

Estudos como o de Völter et al.^(16)^, que investigaram a versão alemã do MoCA-H, demonstram que o instrumento é adequado para pessoas com perda auditiva, apresentando alta confiabilidade teste-reteste e resultados semelhantes entre dois grupos, sendo um com audição normal ou perda auditiva de grau leve, e outro com perda auditiva de grau moderado a profundo, ambos os grupos sem declínio cognitivo. Esses achados reforçam a importância de validar e aplicar o MoCA-H em diferentes línguas e culturas, a fim de garantir que a avaliação leve em consideração os impactos culturais e linguísticos^(18)^ e seja efetivamente centrada na cognição, e não afetada por fatores sensoriais.

Diante disso, a presente pesquisa justifica-se pela necessidade de investigar se o MoCA-H no português brasileiro consegue avaliar de maneira equivalente o desempenho cognitivo de idosos com e sem perda auditiva tratada, contribuindo para a validação funcional do instrumento e promovendo maior precisão nas triagens cognitivas em contextos clínicos audiológicos. O objetivo deste estudo, portanto, foi analisar o desempenho do MoCA-H no português brasileiro em dois grupos de idosos neurologicamente saudáveis: um grupo com média quadritonal dentro dos padrões de normalidade e outro com perda auditiva previamente diagnosticada e usuários de próteses auditivas. Com base nisso, espera-se que ambos os grupos apresentem desempenho semelhante nas habilidades avaliadas pelo MoCA-H e no escore total do instrumento, uma vez que são neurologicamente saudáveis e não apresentam queixas cognitivas.

## MÉTODO

Trata-se de uma pesquisa de caráter observacional, transversal e quantitativo, com amostra selecionada por conveniência, conduzida de acordo com as diretrizes e normas regulamentadoras para pesquisas envolvendo seres humanos, conforme estabelecido na Resolução 466/12, do Conselho Nacional de Saúde, e aprovada pelo Comitê de Ética e Pesquisa (CEP) da instituição onde foi desenvolvido o estudo, sob o número 5.162.650.

A pesquisa atual foi desenvolvida pelo mesmo grupo de pesquisadores que realizou a adaptação transcultural do Montreal Cognitive Assessment - Hearing Impairment (MoCA-H)^(18)^, bem como as etapas de validação de critério e de construto do instrumento^(19)^. Todos os indivíduos que consentiram em participar do estudo assinaram o Termo de Consentimento Livre e Esclarecido (TCLE), no qual estavam descritos o objetivo da pesquisa, os procedimentos aos quais seriam submetidos, assim como os riscos e benefícios envolvidos.

Os participantes foram captados por meio da divulgação da pesquisa em redes sociais, grupos de convivência familiar e profissional, além da participação de usuários já acompanhados no ambulatório de prótese auditiva vinculado a uma universidade pública e em uma clínica privada no interior do estado do Rio Grande do Sul (RS).

Os critérios de elegibilidade para o estudo foram: idade igual ou superior a 60 anos; ausência de sinais de declínio cognitivo, detectados por meio da aplicação do Mini Exame do Estado Mental - MEEM^(20)^; ausência de doenças neurológicas prévias autorrelatadas; tempo de estudo formal de, no mínimo, quatro anos; acuidade visual para perto preservada ou corrigida e, média quadritonal (0,5kHz, 1kHz, 2kHz e 4kHz) dentro da normalidade (<20dB) ou perda auditiva de grau moderado ou superior, bilateralmente^(3)^. Para os sujeitos com perda auditiva, foram acrescidos os seguintes critérios de inclusão: estar adaptado com Aparelhos de Amplificação Sonora Individual (AASI), de acordo com o indicado para cada caso, há pelo menos três meses e fazer uso dos dispositivos por, no mínimo, seis horas por dia (averiguado por meio do datalogging); e, ter compreensão de fala em conjunto aberto, ou seja, ser capaz reconhecer a palavra exclusivamente por meio da audição. Foram excluídos os sujeitos que não concluíram todas as etapas preconizadas pelo estudo ou que impossibilitaram o emparelhamento entre os grupos, considerando as variáveis idade e escolaridade.

A partir dos critérios de elegibilidade, foram selecionados 30 participantes: 15 com média quadritonal dentro dos padrões de normalidade e 15 com perda auditiva bilateral de grau moderado ou superior (média quadritonal igual ou superior a 35 dB, conforme a OMS, 2020). Desses, dez foram excluídos devido à presença de dados discrepantes em relação às variáveis definidas para o emparelhamento.

A amostra foi inicialmente dividida, conforme critério metodológico previamente definido, em dois grupos distintos: Grupo Sem Perda Auditiva (GSPA) e Grupo Com Perda Auditiva (GCPA), sendo o primeiro composto por idosos com média quadritonal dentro da normalidade (<20dB) e o segundo por pessoas que apresentavam perda auditiva de grau moderado ou superior^(3)^

Após essa divisão, os grupos foram emparelhados por idade e escolaridade, a fim de garantir comparabilidade entre eles e minimizar possíveis vieses relacionados a essas variáveis. Assim, a amostra final foi composta por 20 idosos, igualmente distribuídos entre os grupos (dez em cada) e entre os sexos (dez do sexo masculino e dez do feminino), com idades variando de 61 a 74 anos (M = 67,5; DP = 3,6) e tempo de escolaridade formal entre quatro e 25 anos (M = 12,4; DP = 5,9) (Quadro 1).

**Quadro 1:** Caracterização da amostra de acordo com as variáveis idade e escolaridade

**Grupo**	**Sujeito**	**Idade**	**Escolaridade (anos)**
**GSPA**	S1	66	15
	S2	64	13,5
	S3	68	17,5
	S4	64	17
	S5	64	17
	S6	65	16
	S7	65	5
	S8	70	21
	S9	70	11
	S10	66	11
**GCPA**	S11	68	13
	S12	67	5
	S13	73	17
	S14	70	8
	S15	73	11
	S16	68	5
	S17	70	8
	S18	61	7
	S19	63	4
	S20	74	25

**Legenda:** GSPA - grupo sem perda auditiva; GCPA - grupo com perda auditiva

A caracterização dos participantes do GCPA, quanto ao tipo e grau da perda auditiva, tempo de adaptação dos AASIs e média de uso diário dos dispositivos, está apresentada no Quadro 2.

**Quadro 2:** Caracterização dos sujeitos do GCPA quanto ao tipo e grau da perda auditiva, bem como quanto ao tempo da protetização e uso dos dispositivos de amplificação

**Sujeito**	**Tipo de PA OD**	**Grau de PA OD**	**Tipo de PA OE**	**Grau de PA OE**	**Tempo de protetização**	**Datalogging h/dia**
S11	PAS	Moderado	PAS	Moderadamente severo	9 meses	14
S12	PAS	Moderadamente severo	PAS	Moderado	4 meses	8
S13	PAS	Moderado	PAS	Moderado	7 meses	11,8
S14	PAS	Severo	PAS	Moderado	3 meses	13
S15	PAS	Moderadamente severo	PAS	Moderadamente severo	6 meses	8
S16	PAS	Moderadamente severo	PAM	Moderadamente severo	3 meses	10
S17	PAS	Severo	PAS	Moderadamente severo	11 meses	7
S18	PAM	Severo	PAM	Severo	10 meses	15,5
S19	PAS	Moderado	PAM	Moderadamente severo	15 meses	6
S20	PAS	Completo	PAS	Moderado	6 meses	6 na melhor orelha^*^

* Tendo em vista que a orelha direita não possuía limiares amplificáveis, a protetização do S20 foi realizada apenas unilateralmente

**Legenda:** GCPA - Grupo com perda auditiva; PA - perda auditiva; PAS - perda auditiva do tipo sensorioneural; PAM - perda auditiva do tipo mista; OD - orelha direita; OE - orelha esquerda

Para a composição amostral, foram realizados os seguintes procedimentos: inspeção do meato acústico externo, audiometria tonal liminar, logoaudiometria, imitanciometria, aplicação do MEEM e anamnese clínica. Após essa etapa inicial, os participantes foram avaliados por meio do MoCA-H. Na anamnese, investigou-se o histórico e tempo da perda auditiva, presença de otites, hábitos como o uso de fones de ouvido, ocupação e exposição ao ruído, antecedentes médicos e familiares (neurológicos ou sindrômicos), uso de medicamentos, possíveis alterações visuais e a escolaridade. Também foram investigados sintomas associados à infecção por COVID-19, incluindo possíveis queixas cognitivas e/ou de memória. Contudo, na ausência de queixas cognitivas consistentes ou de histórico familiar relevante, esses relatos não foram aprofundados, uma vez que não havia indícios clínicos que justificassem uma investigação mais detalhada.

Sendo assim, para a avaliação das funções cognitivas e com o objetivo de definir a população do estudo, utilizou-se o MEEM. Durante a aplicação deste instrumento, todos os participantes com perda auditiva estavam utilizando Aparelho de Amplificação Sonora Individual (AASI) e foram empregadas estratégias de compensação (fala articulada, de frente para o sujeito, em um ambiente silencioso e bem iluminado) sempre que necessário, uma vez que sua aplicação ocorre de forma oral. O protocolo possui um total de 30 pontos, com padrões de normalidade baseados na escolaridade: 21 pontos - analfabetos; 22 pontos - baixa escolaridade (um a cinco anos); 23 pontos - média escolaridade (seis a 11 anos); 24 pontos - alta escolaridade (12 anos ou mais)^(21)^.

Por fim, o MoCA-H foi aplicado por uma das duas pesquisadoras devidamente treinadas para conduzir e interpretar o instrumento, garantindo padronização e confiabilidade nos procedimentos. O MoCA-H avalia as funções cognitivas por meio de oito habilidades: habilidades visuo-espacial/executiva, nomeação, memória, atenção, linguagem, abstração, evocação tardia e orientação.

A habilidade “visuo-espacial/executiva” apresenta três tarefas, que totalizam cinco pontos: uma avalia flexibilidade cognitiva e inibição (funções executivas), por meio de um teste de trilha alternada (escore = 1); e duas tarefas visuo-espaciais: cópia de um cubo (escore = 1) e desenho de um relógio (escore = 3). A “nomeação” é avaliada no teste a partir da evocação de três desenhos de animais, sendo que cada desenho corresponde a um ponto (escore = 3). A “memória” é avaliada a partir de uma lista contendo cinco palavras (rosto, veludo, rosas, igreja e azul), com instrução para que o indivíduo as repita em duas tentativas, mesmo que obtenha êxito na primeira. Neste momento, não há pontuação atribuída. A “atenção” é avaliada por meio da repetição de dígitos em ordem direta e inversa (escore = 2), cálculo mental (escore = 3) e vigilância (escore = 1). A habilidade “linguagem” é avaliada por meio de duas tarefas: a fluência verbal, na qual o participante deve produzir palavras que iniciem com a letra F em um minuto (igual ou superior a 11 palavras, escore = 1), e a reorganização de palavras para a construção sentença, sendo que cada frase correta corresponde a um ponto (escore total = 2). O domínio “abstração” é avaliado por meio da categorização de pares de palavras (escore = 2). A habilidade “evocação tardia” consiste na recordação da lista de palavras apresentada anteriormente na habilidade “memória” (rosto, veludo, rosas, igreja e azul), sendo que cada palavra corretamente evocada corresponde a um ponto (escore total = 5). Por fim, o domínio “orientação” é avaliado pela capacidade do participante de informar a data (escore = 1), mês (escore = 1), ano (escore = 1), dia da semana (escore = 1), local (escore = 1) e cidade (escore = 1) em que se encontra, totalizando 6 pontos.

Para aplicação deste instrumento foram apresentados 77 cartões (disponibilizados em uma folha A4, na posição horizontal), contendo as atividades que compõem o teste. A partir disso, o participante realizava a leitura de cada cartão em voz alta e deveria seguir as instruções contidas nele. Nas três primeiras tarefas do teste o indivíduo foi solicitado, por meio das orientações contidas nos cartões, a resolver manualmente algumas tarefas, sendo neste momento entregue a ele a folha do teste e uma caneta. O tempo utilizado para a aplicação do protocolo foi de 20 a 30 minutos, variando conforme dificuldades relacionadas à escolaridade dos sujeitos e hábitos de leitura e escrita, as quais não foram formalmente avaliadas, apenas autorreferidas pelos participantes. Mesmo assim, não houve qualquer tipo de auxílio na leitura por parte das pesquisadoras, uma vez que essa intervenção não é prevista no procedimento. Ressalta-se que, em hipótese alguma, deve-se interferir na aplicação do instrumento. As avaliações foram realizadas individualmente, em salas silenciosas, em um único encontro de, aproximadamente, duas horas.

Os dados da avaliação foram tabulados e as análises estatísticas foram conduzidas no software Jamovi (versão 2.6.45.0). A normalidade das variáveis foi verificada pelo teste de Shapiro–Wilk. A maioria das variáveis apresentou distribuição não normal (p < 0,05), exceto o MoCA Total. Assim, foi utilizado o teste não paramétrico Mann–Whitney para comparações entre grupos. Para a variável MoCA total utilizou-se o teste paramétrico t de Student. O nível de significância adotado foi de 5%.

## RESULTADOS

A Tabela 1 apresenta as pontuações médias de desempenho nas diferentes tarefas do MoCA-H, comparando os resultados obtidos pelos participantes do grupo com perda auditiva com os do grupo sem perda auditiva.

**Tabela 1:** Comparação das habilidades avaliadas no MoCA-H entre os grupos com e sem perda auditiva

**Tarefa/escore máximo**	**Grupo**	**N**	**Média**	**Desvio Padrão**	**p-valor**
visuo-espacial/executiva/5	GCPA	10	2,7	0,8	0,012
	GSPA	10	3,9	1,1	
Nomeação/3	GCPA	10	2,6	0,5	0,681
	GSPA	10	2,7	0,5	
Memória	GCPA	10	7,4	1,3	0,331
	GSPA	10	8,1	1,2	
Atenção/6	GCPA	10	3,8	1,4	0,436
	GSPA	10	4,3	1,5	
Linguagem/3	GCPA	10	1,4	1,2	0,081
	GSPA	10	2,3	0,9	
Abstração/2	GCPA	10	0,8	0,9	0,468
	GSPA	10	1,1	0,9	
Evocação Tardia/5	GCPA	10	2,3	1,4	0,482
	GSPA	10	2,7	1,6	
Orientação/6	GCPA	10	5,8	0,4	0,185
	GSPA	10	5,5	0,5	
MoCA-H Total/30	GCPA	10	20,8	2,6	0,123
	GSPA	10	23,1	4,2	

Teste estatístico: Teste t de student e Mann-Whitney

**Legenda:** GCPA - Grupo com perda auditiva; GSPA - Grupo sem perda auditiva

A partir dos resultados (Tabela 1) observou-se que, de modo geral, ambos os grupos apresentaram desempenho semelhante nas tarefas que compõem o MoCA-H, sem diferenças estatisticamente significativas na maioria das habilidades avaliadas (nomeação, memória, atenção, linguagem, abstração, evocação tardia e orientação), inclusive no total do MoCA-H. A única diferença significativa foi identificada no domínio visuo-espacial/executivo (p = 0,012), no qual o GCPA apresentou média inferior (M = 2,7; DP = 0,8) em relação ao GSPA (M = 3,9; DP = 1,1).

## DISCUSSÃO

A etapa de análise de desempenho teve como objetivo verificar o funcionamento dos itens do instrumento estudado entre os dois grupos, com a expectativa de que apresentassem resultados semelhantes. Considerando que, nesse momento da pesquisa, os participantes não deveriam apresentar indícios de declínio cognitivo identificados pelo MEEM, os grupos foram organizados de modo a manter equivalência nas variáveis de idade e escolaridade, diferindo essencialmente quanto à presença ou ausência de perda auditiva.

Estabeleceu-se, ainda, como critério de elegibilidade para o grupo com perda auditiva o uso efetivo dos aparelhos de amplificação sonora individual (AASI). A utilização dos AASIs é reconhecida por promover uma melhor adaptação sensorial e integração de informações auditivas, reduzindo a carga cognitiva exigida para a compreensão de estímulos linguísticos^(22)^. Esses benefícios ocorrem, em parte, porque os aparelhos de amplificação minimizam o esforço auditivo necessário para decodificar a fala, permitindo que os recursos cognitivos sejam direcionados de forma mais eficiente ao processamento e entendimento das informações recebidas^(23,24)^.

O uso regular de próteses auditivas tem sido associado a uma menor taxa de deterioração cognitiva ao longo do tempo, especialmente quando comparado a indivíduos que não tratam a perda auditiva^(25)^. Além disso, estudos indicam que o uso desses dispositivos contribui para a manutenção de funções cognitivas específicas, como a memória de trabalho e a atenção, reforçando seu papel na preservação da cognição em idosos^(26)^.

Nesse contexto, os grupos de idosos com e sem perda auditiva apresentaram desempenhos semelhantes em sete dos oito domínios avaliados pelo MoCA-H. Esse achado era esperado, uma vez que o instrumento foi especificamente adaptado para indivíduos com deficiência auditiva, eliminando a necessidade de compreensão auditiva durante sua aplicação e, assim, reduzindo o impacto da perda auditiva na avaliação das funções cognitivas^(13,14)^.

De maneira semelhante, um estudo prévio que aplicou o MoCA-H em usuários de aparelhos auditivos demonstrou que a adaptação visual do teste diminui a influência da perda auditiva sobre o desempenho cognitivo, possibilitando uma identificação mais precisa de déficits cognitivos^(17)^. Esse resultado pode ser explicado pelo fato de que a utilização do MoCA-H neutraliza um dos principais vieses da avaliação cognitiva: a sobrecarga perceptiva auditiva e a dificuldade em compreender comandos verbais apresentados oralmente^(10)^.

No entanto, apesar da similaridade no desempenho global entre os grupos, constatou-se diferença significativa na tarefa visuo-espacial/executiva, exigindo uma investigação mais aprofundada sobre os fatores associados a esse resultado. Essa constatação corrobora os achados da adaptação alemã do instrumento, que também identificou diferenças significativas nesta habilidade ao comparar dois grupos: um de indivíduos com limiares dentro dos padrões de normalidade até perda auditiva de grau leve e outro de indivíduos com perda auditiva de grau moderado a profundo^(16)^. Esses resultados sugerem que a perda auditiva pode impactar negativamente no domínio visuo-espacial/executivo.

Em relação às possíveis justificativas para a diferença constatada, destaca-se que, em idosos com perda auditiva, há evidências de alterações estruturais e funcionais no cérebro que podem afetar habilidades cognitivas visuo-espaciais e executivas. O estudo de Kirschen e Leaver^(27)^ utilizou neuroimagens para mostrar que limiares auditivos mais elevados estão associados à redução do volume de substância branca em regiões temporais e parahipocampal, alterações na espessura cortical e expansão ventricular, sugerindo reorganização ou degeneração cerebral relacionada à perda auditiva. Além das alterações volumétricas, a perda auditiva pode induzir deaferentação neuronal e reorganização funcional do córtex auditivo. Dietrich *et al.* ^(28)^ demonstraram, por meio de magnetoencefalografia em adultos com perda auditiva em altas frequências, que a deaferentação resultante de danos cocleares promove reorganização cortical nas áreas auditivas, alterando padrões de ativação funcional e recrutamento de regiões adjacentes. Em um estudo mais recente, Tong *et al.* ^(29)^ utilizaram técnicas avançadas de conectividade funcional para mapear o gradiente funcional do conectoma cortical em idosos com perda auditiva relacionada à idade, evidenciando redistribuição de atividade em redes sensoriais e cognitivas superiores, sugerindo que a reorganização cortical não se restringe às áreas auditivas, mas envolve alterações integradas em múltiplas redes neurais. Esses achados indicam que a deaferentação auditiva pode impactar a eficiência de redes cerebrais associadas a funções cognitivas complexas, como processamento visuo-espacial e execução de tarefas cognitivas de ordem superior.

Além disso, ao comparar imagens de ressonância magnética entre indivíduos com perda auditiva e indivíduos com audição normal, Lin *et al*. ^(30)^ identificaram maior redução do volume total do cérebro e também maior redução do volume do lobo temporal direito entre os indivíduo com perda auditiva, reforçando a associação entre déficits auditivos e alterações estruturais cerebrais. Corroborando esses achados, um estudo de coorte prospectivo conduzido por Armstrong *et al.* ^(31)^ apontou a perda auditiva adquirida na vida adulta como um fator de risco para a redução volumétrica do lobo temporal em idades mais avançadas. Considerando que o lobo temporal é responsável por funções essenciais, como processamento de linguagem, memória, processamento auditivo e habilidades visuais, incluindo reconhecimento de padrões visuais, rostos e objetos familiares, essas alterações estruturais podem ter implicações significativas para as habilidades visuo-espaciais e oculomotoras do indivíduo^(32)^. Também, por conta disso, indivíduos sem perda auditiva tendem a apresentar melhor desempenho em habilidades visuo-espaciais e funções executivas, possivelmente devido à integridade do sistema auditivo, do lobo temporal e das vias neurais, já que a audição desempenha um papel crucial no desenvolvimento e manutenção das funções cognitivas, atuando como uma via de entrada fundamental para estímulos ambientais que promovem a plasticidade neural e o funcionamento cognitivo eficiente^(33)^.

Outra possível hipótese para o menor desempenho dos participantes com perda auditiva na tarefa visuo-espacial/executiva pode estar relacionada à ordem de aplicação das atividades no MoCA-H. Considerando que o desempenho inferior foi observado apenas na primeira tarefa, é possível que a ansiedade ou a excitação inicial tenha influenciado negativamente nos resultados. Nesse contexto, a Lei de Yerkes-Dodson pode ser aplicada, uma vez que ela descreve como níveis elevados de ativação emocional no início de uma tarefa podem prejudicar o desempenho, especialmente em atividades que exigem maior controle cognitivo^(34)^.

De acordo com a teoria, o início de uma avaliação com tarefas mais desafiadoras eleva os níveis de excitação do indivíduo a ponto de ultrapassar o ponto ótimo de desempenho, o que resulta em uma execução abaixo do esperado. A ansiedade gerada pela necessidade de se adaptar rapidamente a uma tarefa mais complexa consome recursos cognitivos e gera uma sobrecarga cognitiva temporária, prejudicando a capacidade de concentração e execução da tarefa de forma eficaz. Isso leva a uma diminuição no rendimento, que pode ser superada nas tarefas subsequentes, à medida que os níveis de excitação se estabilizam^(35)^, embora este efeito possa ser observado de maneira geral em diferentes populações, não sendo exclusivo de indivíduos com perda auditiva.

Além disso, a complexidade destas tarefas também pode ser um fator influente, visto que paralelamente ao processo do surgimento de alterações auditivas relacionadas ao envelhecimento, os idosos frequentemente enfrentam dificuldades adicionais em tarefas cognitivas complexas, devido ao aumento da carga cognitiva necessária para compensar a perda sensorial^(7,36)^. O declínio nas funções cognitivas pode resultar em menor disponibilidade de recursos para o desempenho em atividades que exigem planejamento, organização, compreensão, resolução de problemas e memória, funções tipicamente associadas às áreas pré-frontais do cérebro^(37)^.

Um possível viés metodológico a ser considerado refere-se ao planejamento da coleta de dados, uma vez que toda a bateria de exames e testes, incluindo anamnese direcionada, leitura e assinatura do TCLE, audiometria tonal liminar, logoaudiometria, imitanciometria, MEEM e MoCA-H foi realizada no mesmo dia. Os protocolos cognitivos MEEM e MoCA-H foram sempre aplicados por último, embora, em alguns casos, tenha havido variação na ordem entre os dois instrumentos. Essa estratégia pode ter levado à fadiga cognitiva dos participantes, o que, por sua vez, pode ter impactado no desempenho nos testes, considerando que estudos recentes indicam que tarefas cognitivas prolongadas podem induzir fadiga mental, afetando negativamente o desempenho cognitivo e físico em adultos mais velhos^(38)^.

Uma limitação adicional refere-se ao perfil educacional dos participantes, cuja média de escolaridade foi de 12,4 anos (DP = 5,9), superior à média nacional para pessoas com 25 anos ou mais em 2024, segundo dados do IBGE^(39)^. Apesar da média nacional ter aumentado de forma consistente, passando de 9,1 anos em 2016 para 10,1 anos em 2024, a escolaridade da amostra do estudo ainda permanece acima dessa média, sendo importante destacar que os resultados encontrados podem não refletir completamente a realidade de idosos com menor escolaridade ou de regiões com perfis educacionais distintos.

Além disso, a aplicação dos testes ao final de uma bateria extensa pode gerar impaciência ou pressa para concluir o procedimento, comprometendo o engajamento e a concentração do participante^(40)^. A possível ansiedade gerada pela duração da avaliação ou pela complexidade das tarefas finais pode também ter influenciado o desempenho, sobretudo na tarefa visuo-espacial/executiva do MoCA-H, que foi a primeira apresentada no protocolo, conforme discutido com base na Lei de Yerkes-Dodson^(35)^. Em suma, a diferença significativa observada na tarefa visuo-espacial/executiva, entre idosos com e sem perda auditiva pode ser explicada por diversos fatores, como a deaferentação neuronal, a ordem das atividades no teste e a complexidade da tarefa.

Considerando o conjunto dos resultados, a ausência de diferenças significativas em sete das oito habilidades avaliadas, assim como no escore total do MoCA-H, sugere que esse instrumento é eficaz para avaliar funções cognitivas em idosos com perda auditiva, minimizando o impacto da deficiência sensorial nos resultados. Esse achado reforça a importância de utilizar instrumentos adaptados, como o MoCA-H, que garantem uma avaliação mais precisa e equitativa do desempenho cognitivo em contextos clínicos e de pesquisa. Ao eliminar as barreiras relacionadas à compreensão auditiva, o MoCA-H permite que as habilidades cognitivas dos idosos sejam avaliadas de maneira mais fidedigna, oferecendo uma ferramenta valiosa para a detecção precoce de comprometimentos cognitivos, sem o viés da perda auditiva.

## CONCLUSÃO

Constatou-se que idosos neurologicamente saudáveis, sem e com perda auditiva tratada, apresentaram desempenho semelhante na maior parte das tarefas do MoCA-H. Apenas o domínio visuoespacial/executivo diferenciou significativamente os grupos avaliados. De modo geral, o MoCA-H demonstrou ser uma ferramenta eficaz e sensível para a avaliação cognitiva de idosos com perda auditiva moderada à severa, ao menos na amostra analisada.
